# The effects of feeding sodium chloride pellets on the gastric mucosa, acid‐base, and mineral status in exercising horses

**DOI:** 10.1111/jvim.16851

**Published:** 2023-09-30

**Authors:** Farina Alshut, Monica Venner, Gunilla Martinsson, Ingrid Vervuert

**Affiliations:** ^1^ Equine Veterinary Clinic Destedt GmbH Destedt Germany; ^2^ Institute of Animal Nutrition, Nutrition Diseases and Dietetics, Faculty of Veterinary Medicine University of Leipzig Leipzig Germany; ^3^ Niedersächsisches Landgestüt Celle Celle Germany

**Keywords:** electrolyte, equine, gastric ulcer, health, sweat, water intake

## Abstract

**Background:**

Electrolyte supplementation may be a risk factor for gastric mucosal lesions, but relevant evidence is limited in horses.

**Hypothesis:**

Investigate the effects of PO sodium chloride (NaCl) supplementation on the gastric mucosa of exercising horses. We hypothesized that NaCl supplementation would neither cause nor exacerbate existing gastric mucosal damage.

**Animals:**

Fifteen 3‐year‐old healthy Warmblood stallions from a stud farm.

**Methods:**

Placebo‐controlled study with a crossover design. Horses were fed either a NaCl pellet at a dosage adequate to replace the electrolyte losses in 10 L sweat or a placebo for 19 days with a washout period of 14 days between treatments. The gastric mucosa was evaluated by gastroscopy before and after treatment. Blood samples were collected for evaluation of acid‐base status, packed cell volume (PCV), and total protein, creatinine and blood urea nitrogen concentrations. Urine was collected, and urine specific gravity, electrolyte, creatinine, and urea concentrations were measured.

**Results:**

The initial prevalence of gastric mucosal lesions was 85%. Sodium chloride pellets did not adversely affect the gastric mucosa and treatment did not significantly alter the hematologic and serum biochemical variables. Urine creatinine concentrations significantly decreased and urinary sodium concentrations significantly increased after supplementation with NaCl pellets. Water intake did not significantly differ between treatments.

**Conclusions and Clinical Importance:**

Daily NaCl pellet supplementation is a palatable and safe way to replace electrolyte losses from sweating in exercising horses and has no negative effects on the gastric mucosa.

**Abbreviations**
BUN: blood urea nitrogen
BW: bodyweight
Ca: calcium
Cl: chloride
EGUS: equine glandular gastric disease
EGGD: equine glandular gastric disease
ESGD: equine squamous gastric disease
HCO_3_^−^
: hydrogen carbonate
*H. pylori*
: *Helicobacter pylori*
K: potassium
Mg: magnesium
Na: sodium
NaCl: sodium chloride
NSAID: nonsteroidal anti‐inflammatory drug
P: phosphorus
pCO_2_
: carbon dioxide partial pressure
PCV, packed cell volume; pO_2_
: oxygen partial pressure
USG: urine specific gravity

## 1 INTRODUCTION

Gastric ulcerations are common in horses. The prevalence reaches 100% in sports horses, especially in racehorses.^1^, ^2^ In nonracing sports horses, the prevalence of gastric ulceration ranges from 11%^3^ to 83.5%.^4^ The suspected risk factors for development of equine gastric ulcer syndrome (EGUS) include training, stress, use of non‐steroidal anti‐inflammatory drugs (NSAIDs), transportation, lack of turnout, and no contact with other horses.^5^, ^6^ Nutritional risk factors for EGUS, mostly equine squamous gastric disease (ESGD), include high starch intake (>2 g/kg body weight [BW]/day), low forage intake, >6 hours fasting between forage feeding, and intermittent water access.^5^, ^7^ Electrolyte supplementation also may increase the risk of gastric lesion formation in horses. A previous study showed that PO administration of a hypertonic solution every hour for 8 hours increased the mean number and severity of gastric ulcers in the squamous gastric region in horses.^8^

Strong clinical evidence supports an association between higher salt and salted food intake and the development of gastritis and stomach cancer in humans, as reviewed previously.^9^ In laboratory rodents, high salt intake has been shown to induce gastritis and gastric epithelial proliferation.^10^, ^11^

Several studies indicate the positive effects of electrolyte supplementation on rehydration and compensation for electrolyte losses in sport horses.^12^, ^13^, ^14^, ^15^ However, little is known about the impact of electrolyte intake on the gastric mucosa in horses except for a single study.^8^ In that study, salt was given in the form of a concentrated paste PO hourly for 8 hours to simulate conditions during an endurance race, which might be the reason for the increased gastric mucosal lesions. Differences in application form and frequency of a palatable salt pellet given together with concentrated feed and divided into several meals throughout the day might be a safer way to compensate for sweat losses in the exercising horse.

Our aim was to investigate the effects of NaCl supplementation as a pellet on the gastric mucosa, as well as acid‐base and mineral status in exercising horses. We hypothesized that PO NaCl pellets at a dosage adequate to replace the salt loss from 10 L of sweat would neither cause nor aggravate existing gastric mucosal damage and would not have negative effects on acid‐base and mineral status in exercising Warmblood horses.

## 2 MATERIALS AND METHODS

### 2.1 Animals and housing

Fifteen 3‐year‐old Warmblood stallions with a mean (±SD) body weight of 554 ± 30 kg were included in the study. The horses were stalled in individual boxes and began to be trained approximately 4 months before study onset. The horses were exercised according to a standardized protocol and rested 1 day per week (File S1). For 2 days per week, the horses had 2 hours of access to a sand paddock. The project design and methodology were approved by the Ethics Committee for Animal Rights Protection of the LAVES (No. TVV 33.19‐42 502‐04‐20/3572) in accordance with German legislation for animal rights and welfare.

### 2.2 Basal diet

All horses were fed hay (1.5 kg fresh matter/100 kg BW), 2850 g oats, and 1350 g compound feed (DERBY Standard; Engelter GmbH, Büttelborn, Germany) divided into 3 daily meals. Ten grams NaCl, 50 g soybean meal, 100 g mineral supplement (DERBY Mineral‐Pellets; Engelter GmbH), and 125 mL flaxseed oil were mixed into the morning meal. The basal diet was fed independently from the treatment pellet (see below). The total amount of sodium (Na) intake in the basal diet was approximately 13.7 g/day, the total amount of chloride (Cl) was approximately 117.8 g/day.

### 2.3 Treatment pellets

The NaCl pellet provided 50 g Na/kg pellet and 65 g Cl/kg pellet. To improve palatability, the NaCl pellets consisted of 60% barley, 15% dried grass, and in amounts below 10%: flaxseed meal, dextrose, corn, sodium bicarbonate, carob, cellulose, sepiolite, fennel seeds, and licorice root powder. The NaCl pellets were administered at a dosage that would effectively compensate for electrolytes in 10 L/day sweat losses. The treatment dose of 600 g/day was calculated to replace salt losses from 10 L sweat (3.1 g Na/L sweat^16^). The salt intake by the pellet included a total of 30 g Na and 39 g Cl per day. The placebo pellets consisted of barley, dried grass, flaxseed meal, corn, carob, dextrose, cellulose, and sepiolite in the same percentages as the NaCl pellets.

### 2.4 Study protocol

**FIGURE 1:**
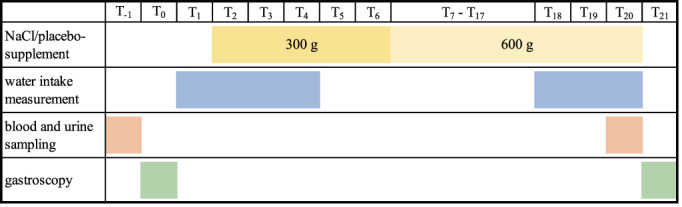
Study protocol overview of a single treatment period (T = day).

The study was performed using a placebo‐controlled crossover design with 2 feeding periods of 19 days and a washout period of 14 days between the feeding periods (Figure 1). Assignment to the groups (NaCl vs placebo) was done randomly before the first feeding period with half of the horses receiving the NaCl pellets and the other half receiving the placebo pellets. In the second feeding period, the groups were switched to the other treatment. Before and after each feeding period, blood was collected from the jugular vein and urine was collected. The horses were then fasted 12 hours overnight and gastroscopy was performed on the next day.

#### 2.4.1 Treatment

The treatment period lasted 19 days. In the beginning, the horses were gradually introduced to the treatment. From day 2 (T_2_) to day 6 (T_6_) horses were fed half of the calculated dose (300 g NaCl pellets or 300 g of the placebo pellets) divided into 2 meals of 150 g, fed in the morning and evening, respectively. From T_7_ to T_20_, all horses were fed 600 g/day of the NaCl or placebo pellet divided into 2 meals of 300 g fed in the morning and evening, respectively. The NaCl or placebo pellets were mixed with oats and compound feed.

#### 2.4.2 Gastroscopy

On days 0 (T_0_) and 21 (T_21_), gastroscopy was performed using a 3‐meter flexible gastroscope (KARL STORZ, Tuttlingen, Germany). The horses were fasted by muzzling for 12 hours and had access to water until 2 hours before the procedure. The horses were sedated with detomidine (Cepesedan; CP Pharma Handlungsgesellschaft mbH, Burgdorf, Germany) at a dosage of 0.014‐0.026 mg/kg BW. The gastric mucosa was evaluated by 2 investigators blinded to the group assignment of each horse. The regions examined included the dorsal squamous fundus, the lesser and greater curvatures of the squamous region, the lesser and greater curvatures of the glandular region, the pyloric antrum, and the pylorus. Each squamous region was scored according to the grading system recommended by the European College of Equine Internal Medicine Consensus Statement (Table 1). A modified scoring system was used for the glandular region (Table 2). In case of an initial score of 4 in 1 or both stomach regions, the horse was excluded from the study.

**TABLE 1:** European College of Equine Internal Medicine grading system for equine squamous gastric disease (ESGD).^5^

Grade	Characteristics
0	Epithelium intact and no appearance of hyperkeratosis
1	Mucosa intact, but areas of hyperkeratosis
2	Small, single, or multifocal lesions
3	Large single or extensive superficial lesions
4	Extensive lesions with areas of apparent deep ulceration

**TABLE 2:** Modified grading system for equine glandular gastric disease (EGGD).^17^

Grade	Characteristics
0	Epithelium intact and no appearance of hyperemia (reddening) or fibrinosupperative areas
1	Intact flat mucosa, but with small single or multifocal areas of reddening
2	Raised mucosa with large single or multifocal areas of reddening or fibrinosupperative areas, no signs of bleeding
3	Raised mucosa with hemorrhagic and fibrinosupperative areas
4	Ridged or depressed mucosa with severe signs of bleeding or with large and distinct fibrinosupperative areas

#### 2.4.3 Water intake

All horses had access to a water bucket with a scale. The bucket was cleaned and filled 4 times per day. Water intake was measured from T_1_ to T_4_ and from T_18_ to T_20_ by reading the scale before refilling the bucket. The filled water buckets were placed in the boxes 1 day before starting the measurements to acclimate the horses. The minimum and maximum ambient temperatures were recorded on each day of water intake measurement. The horses had access to automatic water dispensers between intake measurement days.

#### 2.4.4 Blood and urine sampling

On T_−1_ and T_20_, blood was taken from the jugular vein via an 18 G needle (SARSTEDT; Nümbrecht, Germany) and collected in serum analysis tubes (Monovetten; SARSTEDT; Nümbrecht, Germany). Syringes containing electrolyte‐compensated dried heparin (Pico; RADIOMETER; Krefeld, Germany) were used for the blood gas analyses.

Blood used in the serum analyses was centrifuged (3000 rpm, 10 min) after 30 minutes clotting and the sera were stored at −20°C until analysis. Urine was taken on T_−1_ and T_20_ by collecting midstream voided urine in a container. Before each urine sample collection, the horses were confined without water on a paddock for 2 hours and urine was collected by spontaneous urination immediately after horses returned to the boxes. The urine samples were stored at −20°C until analysis. Urine could not be collected from all horses. Consequently, less data for urine were available than for blood and gastric variables (Table 6).

### 2.5 Analysis

#### 2.5.1 Scaling

Body weight was measured using an electronic scale (FX1 Weight System; Texas Trading, Windach, Germany) on T_−1_ and T_20_.

#### 2.5.2 Clinical examination

Each horse was clinically examined before and after each feeding period on T_0_ and T_21_. The clinical examination included measurements of heart rate, respiratory rate, rectal temperature and parameters of circulation such as color and moistness of mucous membranes and capillary refill time.

#### 2.5.3 Blood and urine analyses

Venous blood pH, oxygen partial pressure (pO_2_), carbon dioxide partial pressure (pCO_2_), hematocrit, hydrogen carbonate (HCO_3_^−^), Na^+^, K^+^, Cl^−^, and ionized calcium (Ca^2+^) concentrations were measured using a RADIOMETER (ABL80 Flex; ABL90; RADIOMETER, Krefeld, Germany) immediately after blood collection. Serum total protein, creatinine, blood urea nitrogen (BUN), Na^+^, K^+^, Cl^−^, Ca^2+^, magnesium (Mg^2+^), and phosphorus (P) concentrations were measured using an automated chemistry analyzer (Roche Cobas C311; Roche Diagnostics GmbH, Mannheim, Germany). Urine specific gravity (USG) was measured on‐site using a hand‐held refractometer. Urinary creatinine, urea, Na^+^, K^+^, Cl^−^, Ca^2+^, Mg^2+^, and P concentrations were measured using the same automated chemistry analyzer used to determine serum concentrations.

#### 2.5.4 Water intake

Mean water consumption was calculated at T_0_ (no treatment), T_1‐3_ (half treatment dose [300 g]), and T_16‐18_ (full treatment dose [600 g]).

### 2.6 Statistical analyses

Data were processed using StatSoft STATISTICA v. 14.0 (TIBCO Software Inc., Palo Alto, California) and SPSS (IBM‐SPSS Statistics 27). A power analysis with a power of 0.95 and a median difference of 1 grade of gastric ulceration between the groups was performed to estimate the minimum required sample size of 14 animals. The data were analyzed for normal distribution using the Shapiro‐Wilk test. Non‐parametric data were tested for significance using the Wilcoxon matched‐pairs test and reported as medians, percentiles (25th and 75th), and ranges. If the data were normally distributed, significance was tested by analysis of variance (ANOVA) and the Fisher least significant difference (LSD) test. Data were reported as mean ± SD. Significance was set at *P* < .05.

## 3 RESULTS

All included animals were clinically healthy based on results of physical examinations conducted at the start and end of the study. Body weights did not change significantly during the study (NaCl: T_−1_:547 ± 29 kg; T_20_: 559 ± 31 kg; placebo: T_−1_:549 ± 31 kg; T_20_: 559 ± 32 kg). None of the horses refused the pellets.

### 3.1 Gastroscopy

Gastric mucosa data are presented in Tables 3 and 4. The overall initial (T_0_) prevalences of gastric lesions were 83.33% according to the total scores for the squamous mucosa (median: 2; minimum: 0; maximum: 3) and 83.33% according to the total scores for the glandular mucosa (median: 1; mininum: 0; maximum: 3; Table 3).

**TABLE 3:** Number of horses with ESGD and EGGD at T_0_ of both treatment periods.

Treatment	Score 0	Score 1	Score 2	Score 3
ESGD 1st period T_0_ Placebo	No Horses: 2 (13.33%)	No Horses: 1 (6.67%)	No Horses: 2 (13.33%)	No Horses: 2 (13.33%)
ESGD 1st period T_0_ NaCl pellets	No Horses: 1 (6.67%)	No Horses: 5 (33.33%)	No Horses: 1 (6.67%)	No Horses: 1 (6.67%)
			*ESGD prevalence*	80%
EGGD 1st period T_0_ Placebo	No Horses: 0 (0%)	No Horses: 4 (26.67%)	No Horses: 3 (20%)	No Horses: 0 (0%)
EGGD 1st period T_0_ NaCl pellets	No Horses: 3 (20%)	No Horses: 2 (13.33%)	No Horses: 3 (20%)	No Horses: 0 (0%)
			*EGGD prevalence*	80%
ESGD 2nd period T_0_ Placebo	No Horses: 1 (6.67%)	No Horses: 2 (13.33%)	No Horses: 2 (13.33%)	No Horses: 3 (20%)
ESGD 2nd period T_0_ NaCl pellets	No Horses: 1 (6.67%)	No Horses: 2 (13.33%)	No Horses: 1 (6.67%)	No Horses: 3 (20%)
			*ESGD prevalence*	86.66%
EGGD 2nd period T_0_ Placebo	No Horses: 1 (6.67%)	No Horses: 5 (33.33%)	No Horses: 2 (13.33%)	No Horses: 0 (0%)
EGGD 2nd period T_0_ NaCl pellets	No Horses: 1 (6.67%)	No Horses: 4 (26.67%)	No Horses: 1 (6.67%)	No Horses: 1 (6.67%)
			*EGGD prevalence*	86.66%

**TABLE 4:** Gastroscopy scores and *P*‐values for each stomach compartment divided by treatment group.

Region	Treatment pellets	T_0_	T_21_	*P*‐value time	*P*‐value treatment T_0_	*P*‐value treatment T_21_
Dorsal squamous fundus	Placebo	0 (0/0); min. 0, max. 0	0 (0/0); min. 0, max. 1	.32	1	.32
	NaCl	0 (0/0); min. 0, max. 0	0 (0/0); min. 0, max. 0	1		
Greater curvature squamous region	Placebo	0 (0/2); min. 0, max. 3	0 (0/2); min. 0, max. 3	.62	1	.41
	NaCl	1 (0/1); min. 0, max. 2	0 (0/2); min. 0, max. 2	.74		
Lesser curvature squamous region	Placebo	2 (1/3); min. 0, max. 3	2 (2/3); min. 0, max. 3	.36	.44	.49
	NaCl	1 (1/3); min. 0, max. 3	2 (1/3); min. 0, max. 3	.35		
Greater curvature glandular region	Placebo	0 (0/0); min. 0, max. 1	0 (0/1); min. 0, max. 2	.1	.56	1
	NaCl	0 (0/0); min. 0, max. 1	0 (0/1); min. 0, max. 1	.26		
Lesser curvature glandular region	Placebo	0 (0/0); min. 0, max. 1	0 (0/0); min. 0, max. 1	1	.71	1
	NaCl	0 (0/0); min. 0, max. 2	0 (0/0); min. 0, max. 1	.71		
Pyloric antrum	Placebo	1 (1/1); min. 0, max. 2	2 (1/2); min. 0, max. 3	.1	1	.58
	NaCl	1 (1/1); min. 0, max. 3	1 (1/2); min. 0, max. 3	.43		
Pylorus	Placebo	0 (0/0); min. 0, max. 2	0 (0/0); min. 0, max. 1	.41	.89	.66
	NaCl	0 (0/0); min. 0, max. 2	0 (0/0); min. 0, max. 2	.71		

*Note*: Data are medians, percentiles (25th/75th), minima and maxima (n = 15).

No findings were detected in the dorsal squamous fundus area except for 1 placebo‐fed horse with score = 1 at T_21_. In the greater curvature of the squamous region, the median lesion scores were 1 (25th percentile: 0/75th percentile: 1) at T_0_ and 0 (0/2) at T_21_ of NaCl treatment (*P* = .74). Before and after placebo, the greater curvature of the squamous region had a median lesion score = 0 (0/2).

In the lesser curvature of the squamous region, the median lesion score was 1 (1/3) at T0. It had increased to 2 (1/3) at T_21_ after the NaCl pellet (*P =* .35). The median lesion scores did not change after placebo feeding. Treatment did not have a significant effect on the median lesion score (T_0_ *P* = .44; T_21_ *P* = .49). The median lesion scores of the greater and lesser curvatures of the glandular mucosa did not differ among time intervals or treatment types.

For the pyloric antrum, the median lesion score of the placebo treated horses increased from 1 (1/1) to 2 (1/2; *P* = .1) and did not change after NaCl pellet treatment. In both feeding groups, the median lesion scores for the pyloric region did not change between T_0_ and T_21_ (placebo: *P* = .41; salt: *P* = .71; treatment: T_0_ *P* = .89; T_21_ *P* = .66).

### 3.2 Blood test results

Blood packed cell volume (PCV), serum total protein concentrations, venous blood pH, and blood pCO_2_ did not change significantly over the course of the study (Table 5). However, blood HCO_3_^−^ concentrations significantly decreased from T_−1_ to T_20_ after placebo (*P* = .01).

**TABLE 5:** Blood values and *P*‐values for placebo and NaCl treatment.

Parameter	Treatment pellets	T_−1_	T_20_	*P*‐value time	*P*‐value treatment T_−1_	*P*‐value treatment T_20_
pH^1^	Placebo	7.39 (7.38/7.41); min. 7.35, max. 7.46	7.39 (7.38/7.40); min. 7.36, max. 7.42	.55	.27	.58
	NaCl	7.38 (7.37/7.40); min. 7.36, max. 7.41	7.40 (7.38/7.41); min. 7.35, max. 7.42	.17		
pCO_2_ (mmHg)^1^	Placebo	52.1 (50.5/54); min. 45.7, max. 57.6	53.2 (51.2/54.2); min. 46.4, max. 58.3	.68	.23	.46
	NaCl	53.6 (52.5/54.2); min. 48.2, max. 56.4	53.3 (51.5/54.6); min. 48.9, max. 58.1	.86		
HCO_3_^−^ (mmol/L)^1^	Placebo	**31** (30.5/31.6); min. 28.2, max. 33.9	**29.7** (28.5/31.2); min. 26.8, max. 32	**.01**	.44	.17
	NaCl	31.1 (29.9/32.1); min. 28.8, max. 33	30.8 (29.4/31.4); min. 28.3, max. 34.7	.37		
PCV (%)^1^	Placebo	42 (39.5/43); min. 37, max. 51	41.5 (40.2/44); min. 37, max. 54.9	.9	.72	.92
	NaCl	42 (40.5/45.5); min. 39, max. 49	41.1 (38/44.1); min. 36.6, max. 54.1	.47		
Serum total protein (g/dL)^2^	Placebo	6.6 (6.4/6.75); min. 5.9, max. 7.8	6.4 (6.25/6.75); min. 5.8, max. 7.2	.18	.65	.53
	NaCl	6.5 (6.4/6.9); min. 6.2, max. 7.8	6.6 (6.35/6.75); min. 6, max. 7	.11		
Na^+^ (mmol/L)^1^	Placebo	**139** (138/140); min. 137, max. 143	**138** (137/139); min. 136, max. 140	**.02**	.42	.51
	NaCl	**140** (139/140); min. 138, max. 141	**137** (136/141); min. 135, max. 141	**.05**		
Na_Total_ (mmol/L)^2^	Placebo	140 (137/141); min. 135, max. 142	138 (137/139); min. 136, max. 141	.28	.31	.5
	NaCl	139 (136/141); min. 131, max. 143	138 (137/140); min. 135, max. 141	.84		
K^+^ (mmol/L)^1^	Placebo	3.35 (3.16/3.88); min. 1.95, max. 5.31	3.5 (3.2/3.6); min. 2.13, max. 3.8	.6	.07	1
	NaCl	**2.99** (2.81/3.3); min. 2.28, max. 3.92	**3.48** (3.17/3.71); min. 2.39, max. 3.85	**.05**		
K_Total_ (mmol/L)^2^	Placebo	3.46 (3.23/3.92); min. 2.26, max. 4.33	3.35 (3.01/3.53); min. 2.63, max. 4.6	.27	.3	.27
	NaCl	3.15 (2.98/3.46); min. 2.49, max. 4.26	3.02 (2.71/3.55); min. 1.94, max. 4.3	.55		
Cl^−^ (mmol/L)^1^	Placebo	94 (94/95); min. 89, max. 98	95 (94.5/96); min. 92, max. 99	.17	.5	.59
	NaCl	95 (94/96); min. 92, max. 97	95 (94/96); min. 92, max. 97	.48		
Cl_Total_ (mmol/L)^2^	Placebo	98.2 (96.5/100.2); min. 93.6, max. 102	98.2 (96.6/98.8); min. 94.4, max. 100.9	.41	.63	.26
	NaCl	97.8 (95.6/100.7); min. 92.2, max. 102.4	98 (96.8/99.7); min. 96.1, max. 101.1	.59		
Ca^2+^ (mmol/L)^1^	Placebo	**1.6** (1.57/1.64); min. 1.5, max. 1.67	**1.55** (1.49/1.61); min. 1.4, max. 1.67	**.04**	.44	.24
	NaCl	**1.59** (1.55/1.61); min. 1.51, max. 1.68	**1.51** (1.47/1.56) min. 1.35, max. 1.61	**.002**		
Ca_Total_ (mmol/L)^2^	Placebo	**2.94** (2.86/3.03); min. 2.69, max. 3.24	**2.76** (2.71/2.82); min. 2.57, max. 2.96	**.002**	.41	.27
	NaCl	**2.87** (2.77/3); min. 2.73, max. 3.14	**2.73** (2.69/2.78); min. 2.61, max. 2.92	**.002**		
P_Total_ (mmol/L)^2^	Placebo	1.23 (1.13/1.35); min. .97, max. 1.64	1.35 (1.24/1.39); min. 1.11, max. 1.49	.16	.18	.27
	NaCl	**1.21** (1.13/1.25); min. .88, max. 1.33	**1.4** (1.26/1.45); min. 98, max. 1.6	**.003**		
Mg_Total_ (mmol/L)^2^	Placebo	**.76** (.74/.8); min. .68, max. .84	**.68** (.65/.71); min. .62, max. .78	**.001**	.46	**.02**
	NaCl	**.76** (.73/.8); min. .65, max. .82	**.64** (.63/.67); min. .6, max. .77	**.001**		
BUN (mmol/L)^2^	Placebo	4.44 (3.83/4.88); min. 2.93, max. 5.9	4.12 (3.79/4.53); min. 2.96, max. 5.54	.36	.77	.33
	NaCl	4.47 (4/4.77); min. 2.62, max. 5.54	4.06 (3.63/4.15); min. 3.03, max. 5.66	.15		
Creatinine (mmol/L)^2^	Placebo	99 (96/106); min. 85, max. 133	100 (95.5/109); min. 89, max. 121	.7	.37	.69
	NaCl	101 (89.5/107); min. 86, max. 127	101 (94.5/108); min. 90, max. 122	.43		

*Note*: Data are medians, percentiles (25th and 75th), and minima and maxima (n = 15). **1**: whole blood parameters, **2**: serum parameters; significant *P*‐values (<.05) are written in bold.

Median blood ionized Na^+^ concentrations decreased significantly in response to both treatments (NaCl: *P* = .05; placebo: *P* = .02). There was no significant difference in blood ionized Na^+^ concentrations between treatments (*P* = .51). Serum total Na_Total_, blood ionized Cl^−^, and serum total Cl_Total_ concentrations did not differ significantly with time or treatment.

Blood ionized Ca^2+^ and serum total Ca_Total_ concentrations significantly decreased (*P* = .002) after NaCl or placebo feedings. Treatment had no effect on temporal changes in blood ionized Ca^2+^ or serum total Ca_Total_ concentrations.

Other blood electrolytes showed some variations over time and treatment but without biological significance (Table 5).

### 3.3 Urine test results

After 19 days of treatment, USG decreased significantly in both groups (NaCl: *P* < .001; placebo: *P* = .01). Nevertheless, treatment had no significant impact on USG (Table 6). Urinary Ca_Total_ concentrations significantly decreased in response to NaCl (*P* = .03) and placebo feeding (*P* = .01) but there were no treatment‐related differences. Urinary Na_Total_ concentrations significantly increased between T_−1_ and T_20_ after NaCl feeding (*P* = .01) but not after placebo feeding (*P* = .85). Urinary Na_Total_ concentrations were significantly higher in the NaCl group than the placebo group at T_20_ (placebo: 21.8 ± 5.6 mmol/L; NaCl: 96.4 ± 61.3 mmol/L; *P* = .01). Urinary Cl_Total_ concentrations did not change significantly or differ with time or treatment. Other urine electrolytes showed some variations including time and treatment effects, but without biological significance.

**TABLE 6:** Urinary values and *P*‐values in response to placebo and NaCl treatment.

Parameter	Treatment pellets	T_−1_	T_20_	*P*‐values		
				Time	Treatment T_−1_	Treatment T_20_
USG	Placebo	**1.047** ± 4.7	**1.038** ± 7.5	**.01**	.39	**.03**
	NaCl	**1.043** ± 7.0	**1.029** ± 6.0	**0**		
Creatinine (mmol/L)	Placebo	**22.4** ± 2.8	**24.3** ± 5.3	.27	.25	**.01**
	NaCl	**19.1** ± 4.5	**15.9** ± 5.8	**.04**		
BUN (mmol/L)	Placebo	**430** ± 129	**349** ± 80.1	**.04**	.54	.76
	NaCl	394 ± 96.5	331 ± 85.1	.06		
Ca_Total_ (mmol/L)	Placebo	**103** ± 26.9	**40.2** ± 6.8	**.01**	.11	.81
	NaCl	**74.1** ± 48.8	**36.1** ± 12.9	**.03**		
Na_Total_ (mmol/L)	Placebo	**17.5** ± 3.7	**21.8** ± 5.6	.85	.55	**.001**
	NaCl	**29.3** ± 15.8	**96.4** ± 61.3	**.01**		
K_Total_ (mmol/L)	Placebo	**259** ± 19.6	**406** ± 45.8	**.003**	.15	**.01**
	NaCl	**313** ± 62.6	**305** ± 90.8	.83		
Cl_Total_ (mmol/L)	Placebo	**296** ± 38	**253** ± 79	>.05	>.05	>.05
	NaCl	**281** ± 42	**312** ± 37	>.05		
Mg_Total_ (mmol/L)	Placebo	**46.5** ± 4.6	**22.2** ± 5.3	**0**	.11	.43
	NaCl	**38.1** ± 13.8	**26.3** ± 6.6	**.01**		
P_Total_ (mmol/L)	Placebo	**0.03** ± .03	**0.10** ± .06			
	NaCl	**0.30** ± .31	**0.36** ± .43			

*Note*: Significant *P*‐values (<.05) are written in bold. Data are means ± SD (placebo: n = 6; NaCl: n = 8). Cl_Total_, insignificant *P*‐values according to ANOVA; P_Total_, changes could not be evaluated as this parameter was below the detection limit in most cases.

### 3.4 Water intake and ambient temperature

Treatment did not change daily water intake (*P* = .89). During placebo treatment, mean water intake was 36.6 ± 10.2 L/day at T_0_, 36.2 ± 4 L/day at T_1‐3_, and 37.7 ± 4.2 L/day at T_18‐20_. During NaCl treatment, mean water intake was 40.2 ± 9.4 L/day at T_0_, 39.2 ± 4.9 L/day at T_1‐3_, and 40.1 ± 3.3 L/day at T_18‐20_. Water intake in relation to ambient temperature is shown in File S2.

## 4 DISCUSSION

Our study was designed to examine the effects of a NaCl supplementation on the gastric mucosa in horses, dosed to replace the loss of NaCl in 10 L sweat. For this purpose, 15 3‐year‐old Warmblood horses received a pelleted supplement along with their compounded feed daily for 19 days. Before the NaCl treatment, the prevalence of gastric mucosal lesions was 83.33%. The NaCl treatment did not have any clinically relevant adverse effects on the gastric mucosa.

The NaCl pellets generally were well accepted and none were left over. Pelleted NaCl feeding is a practical form of electrolyte supplementation and a superior alternative to table salt and salt blocks, because voluntary intake of the latter is usually low.^18^

Interestingly, the prevalence of gastric mucosal lesions with a total overall score ≥1 was already 83.33% in both squamous and glandular regions at the start of the study, but with relatively low scores (Table 3). The median lesion scores were 2 for ESGD and 1 for EGGD, respectively. The high total prevalence observed accorded with results reported in earlier studies. In 1 study, 83.5% of nonracing Danish pleasure horses with unequal workloads (pasture only, light or hard work) had EGUS scores ≥1.^4^ Nevertheless, studies on the prevalence of EGUS in nonracing horses have had equivocal results. One study reported a prevalence of nonglandular gastric lesions (ESGD) of only 11% in riding horses with different riding disciplines.^3^ Horses of different breeds and in the age range of 2 to 23 years were included in that study and the prevalence of nonglandular gastric lesions was higher in horses aged 2 to 6 years (21.4%) compared to the overall prevalence. In our study, all horses were only 3 years old, which might have led to relatively higher prevalences. However, 2‐year‐old Thoroughbred racehorses in training had the largest increase in mean maximum lesion scores compared with those aged 3 years and older.^1^ In a necropsy study on 3715 horses of various breeds aged >1 year, the number of gastric lesions decreased with age especially in Standardbred horses.^19^

Several other factors may have had an impact on the prevalences found in our study. The daily concentrate intake was >4 kg and oats comprised the main feed (2850 g/day). Different studies suggested that the amount of grain fed is an important contributing factor in ESGD development.^7^, ^20^, ^21^ Furthermore, the horses in our study only had paddock access 2 days per week and for 2 hours each time. This condition also might have influenced the observed prevalence of ulcerations in both squamous and glandular gastric regions. However, other studies reported equivocal effects of paddock turnout on ulcer development.^22^, ^23^ One study suggested that time periods above 6 hours between forage feedings could increase lesion scores in the squamous region of the stomach to a greater extent than intervals <6 hours between forage feedings.^7^ In the former case, there is less buffering effect of salivary bicarbonate on stomach acid. The horses in our study usually were subjected to intervals of >10 hours between hay feedings. This management strategy might partially account for the relatively high prevalence of lesions in the squamous regions of the stomachs of the horses. In addition, the study population consisted of stallions, which are predisposed to the development of gastric ulcerations as reported previously.^19^

Electrolyte supplementation provides beneficial effects in maintaining physiological acid‐base and water balance in exercising horses. It increased water consumption and decreased net electrolyte loss in exercising horses and in a furosemide‐induced dehydration model in horses.^12^, ^13^, ^14^, ^15^ It also promoted muscle glycogen resynthesis in horses after exercise.^24^ Most feed rations do not meet the Na and Cl requirements of sweating horses.^16^ Hence, safe electrolyte supplementation strategies must be determined because voluntary NaCl intake is quite variable in horses.^18^

Consistent with our hypothesis, no significant changes were observed in the gastric mucosa regions of the horses fed NaCl pellets for 19 days consecutively. In contrast, a previous study reported a significant increase in the median lesion scores for the gastric squamous regions in horses given 56.7 g hypertonic electrolyte solution (11 056 mg Na; 23 772 mg Cl; 7314 mg K; 1508 mg Ca; and 306 mg Mg) PO q1h for 8 hours consecutively.^8^ The hypertonic solution administration method and quantity used in that study were designed to simulate conditions in endurance racing. Hourly electrolyte paste supplementation is commonly performed during competitions to compensate for electrolyte loss and manage rehydration. However, this high frequency of electrolyte administration over a short time period might have exacerbated the effects of NaCl on the gastric mucosa. In our study, however, the horses received NaCl pellets (30 g Na plus 39 g Cl/horse/day) only twice daily.

There is strong clinical evidence for an association between higher salt and salted food intake and the development of gastritis and stomach cancer in humans, as previously reviewed.^9^ In laboratory rodents, high salt intake has been shown to induce gastritis and gastric epithelial proliferation.^10^, ^11^ In comparison, studies in species other than horses used an estimate of 7.5% to 12.5% NaCl in the diet, either as a single application or as total daily intake. Our study used a pellet 11.5% NaCl, which, together with the amount in the basal diet, translated to approximately 1.2% NaCl in the total diet. This amount is substantially less than in studies of other species.

Our findings suggest that NaCl supplementation in the form of pellets had no negative effect on the gastric mucosa when fed for 19 consecutive days. With respect to the gastric mucosa, it is a safe, convenient, and effective way to replace electrolyte losses in horses. We speculate that even a longer supplementation period of NaCl pellets would not harm the gastric mucosa. The additional NaCl supplementation necessitates a free‐choice supply of water to ensure voluntary water intake by horses.

The positive effects of NaCl supplementation on rehydration and compensation for electrolyte loss have been demonstrated repeatedly.^12^, ^13^, ^14^, ^24^ In contrast, another study reported mild metabolic acidosis in horses receiving NaCl supplementation.^25^ The authors supplemented moderately exercising horses with 100 g NaCl daily for 21 days. After 15 days, however, blood pH significantly decreased from 7.414 to 7.406. As a rule, however, a blood pH of 7.406 is not considered acidotic (normal pH range for horses: 7.38‐7.44).^26^ In our study, no significant changes in blood pH were observed after 19 days of NaCl treatment at 30 g Na/day plus 39 g Cl/day.

Another difference between our study and the previous study^25^ was the amount of forage fed. In the previous study, a low‐forage diet (1 kg/100 kg BW/day) was fed with the expectation that large quantities of hay could compensate for the impact of acidifying agents on blood pH.^27^ In our study, the hay intake rate was 1.5 kg/100 kg BW/day. Nonetheless, it is generally recommended to provide forage at ≥1.5 kg/100 kg BW/day based on dry matter intake.^28^ Low‐forage diets should be avoided because they may cause behavioral, gastrointestinal, and muscular disorders in horses.^5^, ^28^

In our study, serum Na^+^ concentration significantly decreased after both placebo and NaCl pellets. However, no significant difference was found between treatments in terms of the relative impact on serum Na^+^ concentration. In contrast, earlier studies showed that different levels of Na intake did not alter serum Na^+^ concentration in horses.^27^, ^29^ In our study, the cause of the decreases in serum Na^+^ concentrations in both treatment groups is not fully understood, but the moderate decrease in serum Na^+^ concentration did not appear to have any clinically relevant impact.

Prior research on water consumption by horses supplemented with salt showed equivocal results. A linear correlation between NaCl and water intake has been reported.^29^ A 2‐fold increase in Na intake (from 50 mg/kg BW to 100 mg/kg BW) in horses increased water intake by 53% and urinary output by 47%.^30^

On the other hand, 2 other studies reported no correlation between NaCl intake and water consumption.^18^, ^25^ In our study, NaCl pellets likewise did not increase water intake in the horses. However, water intake measurements and outcome were limited because of the switch between automatic water dispensers and water buckets during the feeding periods. It has been shown that horses prefer water from buckets rather than from automated dispensers.^31^, ^32^, ^33^ Here, water provision from a bucket during the feeding periods likely increased water intake despite salt intake. Also, the baseline daily water intake (T_0_) was a single time point, which may not have been an accurate measure, compared with the post‐treatment measures with an average of 3 days (T_1‐3_ and T_18‐20_). Nevertheless, NaCl treatment did not have a significant effect on water intake. We speculate that the effect of NaCl supplementation on water intake was weaker than the effect of water provision by buckets.

The decrease in USG also may reflect changes in water intake associated with water provision by buckets. Hence, the latter might have masked the effects of NaCl treatment. In our study, USG decreased over time in both the NaCl and placebo treated horses but was not influenced by treatment. In contrast, USG significantly decreased from 1.034 to 1.027 after 15 days of feeding 100 g NaCl.^25^ Another study reported a significant decrease in urinary osmolality and significant increase in urine production after PO administration of 0.5 g NaCl/kg BW after dehydration induced by furosemide administration and withholding water for 12 hours.^15^ Additionally, water intake significantly increased during the rehydration phase of horses receiving NaCl compared with those recieving a placebo.^15^

Another limitation of our study was that the ambient temperature differed by approximately 10°C between trials and might have masked the effects of NaCl treatment on water intake. To be able to detect an effect of NaCl on water intake in future research, differences in ambient temperature and changes between types of water provision must be avoided. Air‐conditioned stabling together with automatic water dispensers with a water meter would be ideal to prevent such limitations.

Urine could not be spontaneously collected from all horses in the study. Nevertheless, urinary Na significantly increased with Na intake in response to the NaCl treatment. Similar findings were reported previously.^18^, ^25^, ^27^ Urinary Cl^‐^ excretion also was significantly higher after NaCl supplementation in the previous studies.^18^, ^25^, ^27^ In our study, although urinary Na^+^ concentrations changed in response to the treatments, urinary Cl^−^ concentrations did not. The reason for this discrepancy is unknown despite the fact that the kidney is the principal route of both Na^+^ and Cl^−^ excretion. Because of the response of the kidney to a higher Na intake, the Na^+^ concentration in urine might be a good tool to assess the adequacy of the dietary intake of Na in performance horses. In our study, 31 g of Na was supplemented to compensate 10 L of sweat. The replacement of Na required for 10 L sweat losses was estimated for practical reasons to investigate the effect of NaCl supplementation by a pellet on the gastric mucosa. Therefore, the high amount of Na^+^ excretion after feeding the NaCl pellet could be a result of oversupply because horses in our study may have had lower sweat losses than estimated.

Serum and urinary Ca^2+^ concentrations decreased significantly in both treatment groups over time. However, treatment did not have a significant effect on Ca^2+^ concentrations. We speculate that the increases in water intake in both feeding groups lowered serum, blood and urinary Ca^2+^ concentrations. Similar results in Ca^2+^ concentrations after NaCl supplementation have been reported previously.^18^ On the other hand, increases in urinary Ca^2+^ concentration after NaCl feeding also have been reported.^25^ The authors associated the observed increase in urinary Ca^2+^ excretion with changes in bone remodeling in response to metabolic acidification induced by NaCl intake. In our study, blood pH remained constant. Therefore, changes in bone metabolism by NaCl feeding remained possible but seemed unlikely.

## 5 CONCLUSIONS

We demonstrated that palatable NaCl pellets, fed together with compound feed and divided into 2 equal meals, effectively and safely compensated for electrolyte losses in sweating horses. In agreement with our hypothesis, NaCl supplementation to compensate for 10 L sweat losses (50 g Na/kg pellet and 65 g Cl/kg pellet) did not negatively affect the gastric mucosa, acid‐base balance, and mineral status of exercising horses. In summary, we recommend supplementation using a palatable NaCl pellet in horses to compensate for electrolyte losses in sweat.

## CONFLICT OF INTEREST DECLARATION

Authors declare no conflict of interest.

## OFF‐LABEL ANTIMICROBIAL DECLARATION

Authors declare no off‐label use of antimicrobials.

## INSTITUTIONAL ANIMAL CARE AND USE COMMITTEE (IACUC) OR OTHER APPROVAL DECLARATION

Approved by the Ethics Committee for Animal Rights Protection of the LAVES (No. TVV 33.19‐42 502‐04‐20/3572) in accordance with German legislation for animal rights and welfare.

## HUMAN ETHICS APPROVAL DECLARATION

Authors declare human ethics approval was not needed for this study.

## Supporting information


**File S1.** Exercise program per week.
**File S2.** Mean water intake in relation to the ambient temperature.

## References

[1] Murray MJ , Schusser GF , Pipers FS , et al. Factors associated with gastric lesions in thoroughbred racehorses. Equine Vet J. 1996;28(5):368‐374. 8894534 10.1111/j.2042-3306.1996.tb03107.x

[2] Begg LM , O'Sullivan CB . The prevalence and distribution of gastric ulceration in 345 racehorses. Aust Vet J. 2003;81:199‐201. 15080440 10.1111/j.1751-0813.2003.tb11469.x

[3] Chameroy KA , Nadeau JA , Bushmich SL , Dinger JE , Hoagland TA , Saxton AM . Prevalence of non‐glandular gastric ulcers in horses involved in a university riding program. J Equine Vet Sci. 2006;26(5):207‐211.

[4] Luthersson N , Hou Nielsen K , Harris P , et al. The prevalence and anatomical distribution of equine gastric ulceration syndrome (EGUS) in 201 horses in Denmark. Equine Vet J. 2009;41(7):619‐624. 19927578 10.2746/042516409x441910

[5] Sykes BW , Hewetson M , Hepburn RJ , Luthersson N , Tamzali Y . European College of Equine Internal Medicine consensus statement – equine gastric ulcer syndrome in adult horses. J Vet Intern Med. 2015;29:1288‐1299. 26340142 10.1111/jvim.13578 PMC4858038

[6] Rende DI , Bowen IM , Brazil TJ , et al. EGGD consensus statement – recommendations for the management of equine glandular gastric disease. UK‐Vet Equine. 2018;2(1):2‐11.

[7] Luthersson N , Hou Nielsen K , Harris P , et al. Risk factors associated with equine gastric ulceration syndrome (EGUS) in 201 horses in Denmark. Equine Vet J. 2009;41(7):625‐630. 19927579 10.2746/042516409x441929

[8] Holbrook TC , Simmons RC , Payton ME , MacAllister C . Effect of repeated oral administration of hypertonic electrolyte solution on equine gastric mucosa. Equine Vet J. 2005;37(6):501‐504. 16295925 10.2746/042516405775314880

[9] Wang X , Terry PD , Yan H . Review of salt consumption and stomach cancer risk: epidemiological and biological evidence. World J Gastroenterol. 2009;15(18):2204‐2213. 19437559 10.3748/wjg.15.2204 PMC2682234

[10] Sørbye H , Svanes C , Stangeland L , et al. Epithelial restitution and cellular proliferation after gastric mucosal damage caused by hypertonic NaCl in rats. Vichows Arch A Pathol Anat Histopathol. 1988;413:445‐455. 10.1007/BF00716993 3140485

[11] Ohgaki H , Szentirmay Z , Take M , Sugimura T . Effects of 4‐week treatment with gastric carcinogens and enhancing agents on proliferation of gastric mucosa cells in rats. Cancer Lett. 1989;46:117‐122. 2752382 10.1016/0304-3835(89)90018-9

[12] Nyman S , Jannson A , Dahlborn K , et al. Strategies for voluntary rehydration in horses during endurance exercise. Equine Vet J. 1996;22:99‐106. 10.1111/j.2042-3306.1996.tb05037.x 8894556

[13] Sosa León LA , Hodgson DR , Carlson GP , Rose RJ . Effects of concentrated electrolytes administered via a paste on fluid, electrolyte, and acid base balance in horses. Am J Vet Res. 1998;59(7):898‐903. 9659559

[14] Düsterdieck KF , Schott HC II , Eberhart SW , et al. Electrolyte and glycerol supplementation improve water intake by horses performing a simulated 60 km endurance ride. Equine Vet J. 1999;Suppl 30:418‐424. 10.1111/j.2042-3306.1999.tb05258.x 10659292

[15] Schott HC II , Axiak SM , Woody KA , et al. Effect of oral administration of electrolyte pastes on rehydration of horses. Am J Vet Res. 2002;63:19‐27. 16206775 10.2460/ajvr.2002.63.19

[16] Coenen M , Vervuert I . Pferdefütterung. 6th ed. Stuttgart, Germany: Thieme; 2020.

[17] Vondran S , Venner M , Vervuert I . Effects of two alfalfa preparations with different particle sizes on the gastric mucosa in weanlings: alfalfa chaff versus alfalfa pellets. BMC Vet Res. 2016;12:110. 27301323 10.1186/s12917-016-0733-5 PMC4908680

[18] Schryver HF , Parker MT , Daniluk PD , et al. Salt consumption and the effect of salt on mineral metabolism in horses. Cornell Vet. 1987;77(2):122‐131. 3568683

[19] Sandin A , Skidell J , Häggström J , Nilsson G . Postmortem findings of gastric ulcers in Swedish horses older than age one year: a retrospective study of 3,715 horses (1924‐1996). Equine Vet J. 2000;32(1):36‐42. 10661383 10.2746/042516400777612044

[20] Vatistas NJ , Sifferman RL , Holste J . Induction and maintenance of gastric ulceration in horses in simulated race training. Equine Vet J. 1999;29:40‐44. 10.1111/j.2042-3306.1999.tb05167.x 10696292

[21] Frank N , Andrews FM , Elliott SB , Lew J . Effects of dietary oils on the development of gastric ulcers in mares. Am J Vet Res. 2005;66:2006‐2011. 16334963 10.2460/ajvr.2005.66.2006

[22] Lester GD , Robinson I , Secombe C . Risk Factors for Gastric Ulceration in Thoroughbred Racehorses. Canberra: Australian Government: Rural Industries Research and Development Corporation; 2008:1‐42.

[23] Husted L , Sanchez LC , Olsen SN , et al. Effect of paddock vs. stall housing on 24 hour gastric pH within the proximal and ventral equine stomach. Equine Vet J. 2008;40:337‐341. 18267880 10.2746/042516408X284673

[24] Waller AP , Heigenhauser GJ , Geor RJ , et al. Fluid and electrolyte supplementation after prolonged moderate‐intensity exercise enhances muscle glycogen resynthesis in Standardbred horses. J Appl Physiol. 1985;106(1):91‐100. 10.1152/japplphysiol.90783.2008 18948447

[25] Zeyner A , Romanowski K , Vernunft A , et al. Effects of different oral doses of sodium chloride on the basal acid‐base and mineral status of exercising horses fed low amounts of hay. PloS One. 2017;12(1):e0168325. 28045916 10.1371/journal.pone.0168325 PMC5207637

[26] Walton RM , Cowell RC , Valenciano AC . Equine Hematology, Cytology, and Clinical Chemistry. 2nd ed.: Hoboken, NJ, USA: Wiley Blackwell; 2021; doi:10.1002/9781119500186.

[27] Stürmer K . Untersuchungen zum Einfluss der Fütterung auf den Säure‐Basen‐Haushalt bei Ponys. DVM Thesis, LMU Munich, Germany. 2005.

[28] Harris PA , Ellis AD , Fradinho MJ , et al. Review: feeding conserved forage to horses: recent advances and recommendations. Animal. 2016;11(6):958‐967. doi:10.1017/S1751731116002469. 27881201

[29] Jansson A , Dahlborn K . Effects of feeding frequency and voluntary salt intake on fluid and electrolyte regulation in athletic horses. J Appl Physiol. 1999;86(5):1610‐1616. 10233125 10.1152/jappl.1999.86.5.1610

[30] Meyer H , Perez H , Gonda Y , et al. Postprandial renal and fecal water and electrolyte excretion in horses in relation to kind of feedstuffs, amount of sodium ingested and exercise. Proc Equine Nutr Physiol Symp. 1987;10:67‐72.

[31] Nyman S , Dahlborn K . Effect of water supply method and flow rate on drinking behavior and fluid balance in horses. Physiol Behav. 2001;73:1‐8. 11399288 10.1016/s0031-9384(00)00432-7

[32] Nyman S , Jansson A , Lindholm A , et al. Water intake and fluid shifts in horses: effects of hydration status during two exercise tests. Equine Vet J Suppl. 2002;34:133‐142. 10.2746/042516402776767213 11902756

[33] Van de Weerd HA , Seaman S , Wheeler K , et al. Preference for artificial drinkers in British native ponies. In: 59th Annual Meeting of the European Association for Animal Production, Wageningen Academic Publishers, Wageningen, the Netherlands; 2008:219.

