# Supplementary material for: The effects of feeding sodium chloride pellets on the gastric mucosa, acid‐base, and mineral status in exercising horses
Source: J Vet Intern Med. 2023 Sep 30;37(6):2552–61. doi: 10.1111/jvim.16851 (PMC10658481; doi:10.1111/jvim.16851)
Supplement: Supplementary file 1 — File S1. Exercise program per week. File S2. Mean water intake in relation to the ambient temperature. [file JVIM-37-2552-s001.pdf]

Supplementary File 1. Exercise program per week

Riding	4 times / week	10 min walk<br>10 – 15 min trot<br>5 – 10 min canter with a low velocity
Longing	2 times / week	5 – 10 min walk<br>10 min trot<br>5 – 10 min canter with a low velocity

Supplementary File 2. Mean water intake in relation to the ambient temperature

	Ambient temperature (mean)	10-13 °C (11.3 °C)	16 °C	19-27 °C (23.7 °C)	20-29 °C (24.3 °C)
Water intake (L)	1. trial	**T_{1-3}**	**T_{18-20}**		
	NaCl	112.69	118.88		
	Placebo	102.36	110		
	2. trial			**T_{1-3}**	**T_{18-20}**
	NaCl			123	121.79
	Placebo			114.13	115.88
